# Does chemotherapy-induced neutropaenia result in a postponement of adjuvant or neoadjuvant regimens in breast cancer patients? Results of a retrospective analysis

**DOI:** 10.1038/sj.bjc.6604094

**Published:** 2007-11-13

**Authors:** M Debled, N Houédé, N Madranges, C Donamaria, A Floquet, M Durand, Louis Mauriac

**Affiliations:** 1 Department of Medical Oncology, Institut Bergonié, Bordeaux, France; 2 Department of Pharmacy, Institut Bergonié, Bordeaux, France

**Keywords:** breast, chemotherapy, adjuvant; neutropaenia, dose intensity

## Abstract

In 2005, 224 patients received adjuvant/neoadjuvant chemotherapy for breast cancer in a single institution according to daily practices. Regimens consisted of epirubicin-based chemotherapy (FEC100, four or six cycles), or three cycles of FEC100 followed by three cycles of docetaxel. An absolute blood count was carried out every 3 weeks, 1–3 days before planned chemotherapy cycle. Overall, 1238 cycles were delivered. An absolute neutrophil count (ANC) <1.5 × 10^9^ l^−1^ before planned chemotherapy was found in 171 cycles. Of these, 130 cycles (76%) were delivered as planned regardless of whether ANC levels recovered, and 41 (24%) were delayed. None of these patients developed a febrile neutropaenia. Haematopoietic support (granulocyte colony-stimulating factor (G-CSF)) was required in 12 cycles. We found that the majority of patients with an ANC <1.5 × 10^9^ l^−1^ before planned chemotherapy received planned doses, without complications and need for G-CSF.

The adjuvant treatment of breast cancer has evolved during the last 30 years. In the middle of the 1970s, Bonadonna *et al* (1995) pioneered the use of adjuvant chemotherapy by establishing the contribution of the CMF regimen (cyclophosphamide, methotrexate, and fluorouracil) to improvements in disease-free (DFS) and overall survival (OS). Successive overviews of the Early Breast Cancer Trialists' Collaborative Group (EBCTCG) (2005) have highlighted several trends, which have been reinforced over time: polychemotherapy is superior to monochemotherapy, anthracycline-based chemotherapy is superior to CMF, and the relative benefit of chemotherapy is independent of menopausal status and axillary lymph nodes involvement. Consequently, the anthracyclines are considered pivotal for adjuvant chemotherapy. Although no benefit of an increase in cyclophosphamide doses has been found (Smith *et al*, 2003), it has been shown that doxorubicin was more effective when used at a dose higher than 30 mg m^−2^ (Budman *et al*, 1998), and that an increase in epirubicin dose intensity significantly improved DFS and OS (Bonneterre *et al*, 2005). Therefore, anthracycline doses were gradually increased, especially for the treatment of node-positive breast cancer. Presently, the use of anthracycline-based regimens extends to high-risk, node-negative patients. Taxanes (paclitaxel and docetaxel) were introduced in the 1990s, and their role in the treatment of node-positive early breast cancer has been widely investigated. To date, major randomised trials have shown a significant improvement in DFS (Citron *et al*, 2003; Henderson *et al*, 2003; Mamounas *et al*, 2005; Martin *et al*, 2005; Roche *et al*, 2006) and OS (Citron *et al*, 2003; Henderson *et al*, 2003; Martin *et al*, 2005; Roche *et al*, 2006), when a taxane is added to an anthracycline in sequential, concomitant, or dose-dense regimens.

Both the increase in anthracycline doses and introduction of taxanes have increased the incidence of myelotoxicity, resulting in a higher incidence of neutropaenia, and then an increased risk of febrile neutropaenia and life-threatening infections. According to the rules established by successive randomised trials, it is a current practice to postpone the next cycle for a week and/or to use lower drug doses below an accepted neutrophil count of 1.5 × 10^9^ l^−1^. This is detrimental to maintaining the dose intensity, and consequently, the efficacy of planned regimens (Weiss *et al*, 1987; Wood *et al*, 1994; Bonadonna *et al*, 1995; Budman *et al*, 1998; Chirivella *et al*, 2006). According to the criteria defined by Bonadonna *et al* (1995), it is assumed that chemotherapy dose intensity below 85% of the planned dose significantly decreases the treatment efficacy. To counterbalance the consequences of a low neutrophil count, the use of granulocyte colony-stimulating factors (G-CSFs) has been extended, limiting the risk of infection and maintaining the chemotherapy dose intensity if its reduction may compromise efficacy (Smith *et al*, 2006).

However, a systematic postponement of chemotherapy of 1 week when the neutrophil count is below 1.5 × 10^9^ l^−1^, or the use of G-CSF are often practised in clinical trials, they are not always consistent with community practices. Thereby, we conducted a retrospective analysis of non-selected breast cancer patients receiving adjuvant or neoadjuvant 100-mg m^−2^ epirubicin-based regimens with or without sequential docetaxel. The purpose of this analysis was to evaluate the feasibility of those regimens in daily practices, and the real incidence of treatment delays and use of haematopoietic support.

## PATIENTS AND METHODS

### Study population and treatment regimens

All non-metastatic breast cancer patients who had received adjuvant or neoadjuvant chemotherapy outside clinical trials in 2005 were reviewed in our institution (Institut Bergonié, Bordeaux, France). The data reviewed and recorded in the hospital file were absolute neutrophil count (ANC) measured at baseline, before next chemotherapy cycle and subsequently in case of cycle delay, date of chemotherapy administration and dose, prescription of G-CSF, and toxicities occurring between cycles. The only exclusion criterion was patients treated in neoadjuvant/adjuvant clinical trials.

Planned treatment regimens consisted of FEC100 (fluorouracil 500 mg m^−2^, epirubicin 100 mg m^−2^, cyclophosphamide 500 mg m^−2^ day 1, every 21 days) for four or six cycles according to axillary lymph nodes involvement, or three cycles of FEC100 followed by three cycles of docetaxel (D) 100 mg m^−2^ (day 1, every 21 days) (Roche *et al*, 2006). In case of overexpression and/or amplification of HER-2, patients received trastuzumab started concurrently with docetaxel (D). Adjuvant endocrine therapy and radiotherapy were started at the end of chemotherapy when indicated.

### Data analysis

According to the standard practices of our institution, a blood count is carried out (outside the hospital) the day before the planned chemotherapy infusion (contingently Friday or Saturday when treatment was planned on Monday). The decision to deliver chemotherapy, to control ANC at the time of patient's hospital entry, or to postpone the next chemotherapy cycle depends on ANC, perceived risk of infectious events, and oncologist experience. Qualitative data were presented as a percentage, and ANC quantitative data were described using mean, median, s.d., and range. The relative dose intensity (RDI) was calculated based on the ratio of the drug doses actually delivered in the originally expected time over the expected dose in the expected time (Ferreira Filho *et al*, 2002).

## RESULTS

### Patient characteristics and treatment protocols

During the year 2005, 224 non-metastatic breast cancer patients were treated in this setting. The median age was 49 years (range: 26–72 years) with 37 patients (16.5%) older than 60 years, and three (1.3%) older than 70 years. Four chemotherapy regimens were delivered: (1) four cycles of FEC100; (2) six cycles of FEC100; (3) three cycles of FEC100 followed by three cycles of docetaxel (D); (4) three cycles of FEC100 followed by three cycles of docetaxel (D) plus trastuzumab. Noteworthy, 90% of FEC100-D regimens were initiated after March 2005 consequently to the results of PACS 01 trial (Roche *et al*, 2006). The distribution of each regimen is summarised in Table 1. Sixty-two patients (27.7%) received chemotherapy in neoadjuvant setting.

Planned doses of chemotherapy were well respected (Table 2). The treatment planned was changed for eight patients (3.6%): six with FEC100-D regimen (severe cutaneous toxicity in three, patient's refusal in two, and hypersensitivity in one); and two with FEC100 because of pancytopaenia in one and digestive toxicity in the other. Forty-six cycles (4.5%) were delayed for more than 7 days in 35 patients (15.6%). In half of the cases, this delay was the result of neutropaenia. A 20–25% dose reduction was applied in 19 cycles (1.9%) and nine patients (4%): five patients received FEC100-D, and four received FEC100 (Table 3).

### Absolute neutrophil count, cycle delay, and relative dose intensity

**Figure 1:**
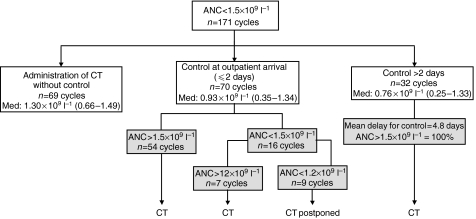
Flow chart of chemotherapy administration according to absolute neutrophil count below 1.5 × 10^9^ l^−1^. ANC, absolute neutrophil count; CT, chemotherapy. Med: median ANC (min-max).

An overall number of 1238 cycles have been administered. Among the 1007 cycles delivered between the second and the sixth courses, ANCs were available in 995 cases (98.8%). The ANCs have been measured on day 21 in 510 cases (51.3%), on day 20 in 136 cases (13.7%), on day 19 in 41 cases (4.1%), and on day 18 in 22 cases (2.2%). In 220 cases (22.1%), ANC was measured at the time of hospital entry for chemotherapy on day 22 or 23. The ANC was measured on days 24–29 in 76 cases (7.6%) because of a cycle delay related to non-haematological toxicities or patient convenience. An ANC <1.5 × 10^9^ l^−1^ was reported in 171 cases (17.2%) secondary to 16 cases out of 220 having an ANC measured on day 22 or 23 (7.2%), 89 out of 510 ANC on day 21 (17.5%), 44 out of 136 ANC on day 20 (32.4%), 15 out of 41 ANC on day 19 (36.6%), and 7 out of 22 ANC on day 18 (31.8%). This situation occurred in 169 cycles after FEC100 out of 791 (21.4%), and in 2 out of 216 cycles (0.9%) after docetaxel (D). Among cycles with ANC <1.5 × 10^9^ l^−1^, three situations were possible (Figure 1). (1) The next chemotherapy cycle was delivered without postponement and no need for a new ANC in 69 cases (40.3%): in seven cases (10.1%) the ANC was below 1 × 10^9^ l^−1^, and in nine cases (13%) the ANC has been measured 3 or 4 days before treatment. (2) A repeat of ANC was measured at the time of patient's hospital entry within 2 days in 70 cases (40.9%). (3) Thirty-two cycles required a repeat of ANC more than 2 days after the first measurement. A new repeat was performed at a mean interval of 4.8 days (range: 3–8 days) showing a neutrophil recovery in 100% of the cases and allowing chemotherapy administration.

Overall, 130 cycles (76%) were delivered as planned with or without a repeat of ANC, and 41 (24%) has to be delayed because of neutropaenia. In case of ANC <1.5 × 10^9^ l^−1^, the subsequent blood counts showed that neutrophil recovery occurred in 75% of cases (39/52) on day +1, in 83% (15/18) on day +2, and in 100% (32/32) from days +3 to +8. When the repeat of ANC has been measured within 48 h following an ANC <1.5 × 10^9^ l^−1^, the neutrophil recovery was 100% (*n*=37) if ANC>1 × 10^9^ l^−1^, and 70% (*n*=33) if ANC<1 × 10^9^ l^−1^.

The RDI of each protocol is described in Table 2. An RDI higher than 95% was reached in 172 patients (76.8%), whereas 14 patients (6.2%) have received lower than 85% of the planned dose. The decrease in RDI was the result of haematologic toxicity or infectious complications in one-third of cases. Other reasons were cycle delays related to patients or hospital convenience.

### Safety profile and haematopoietic support

Among the 76 chemotherapy cycles delivered in spite of an ANC <1.5. × 10^9^ l^−1^, no case of infections were reported. On the other hand, 14 cases of febrile neutropaenia were reported, of which 10 occurred consequently to the first cycle, and four between the second and the sixth cycles with an ANC at the initiation of chemotherapy of 3.51, 3.53, 6.09, and 7.83 × 10^9^ l^−1^, respectively. A secondary prophylaxis with G-CSF was required in eight patients (3.6%) because of febrile neutropaenia in three cases and prevention of neutropaenia-related cycle delay in five cases. The main toxicities of treatment are presented in Table 3. Besides, seven patients (3.1%) experienced a severe cutaneous toxicity related to docetaxel. No toxic death or persistent toxicity was registered.

## DISCUSSION

Over time, growing evidence has emerged that chemotherapy RDI is a key principle of adjuvant chemotherapy efficacy. The long-term follow-up results of the CMF trial, conducted by Bonadonna *et al* (1995), showed that the 20-year DFS of patients was 52% when they had received at least 85% of the planned dose *versus* 27% if the dose was below this rate. This has been confirmed by other retrospective studies using either CMF (Mayers *et al*, 2001) or anthracycline-based regimens (Chirivella *et al*, 2006). Moreover, numerous retrospective studies suggested that early breast cancer patients who experienced the greater myelosuppression related to adjuvant chemotherapy have a trend towards a better outcome (Saarto *et al*, 1997; Colleoni *et al*, 1998; Poikonen *et al*, 1999; Mayers *et al*, 2001; Cameron *et al*, 2003). Additionally, prospective studies demonstrated that an increase in chemotherapy RDI resulted in an improvement of DFS and OS (Budman *et al*, 1998; Citron *et al*, 2003; Bonneterre *et al*, 2005). In spite of these clear data, several retrospective analyses evaluating adjuvant chemotherapy RDI in daily practices have shown a significant decrease in RDI, irrespective of country and chemotherapy regimen (Link *et al*, 2001; Morrow *et al*, 2002; Ottevanger *et al*, 2002; Leonard *et al*, 2003; Lyman *et al*, 2003; Schaapveld *et al*, 2004; Chirivella *et al*, 2006; Shayne *et al*, 2006). Among these analyses, 20–30% of patients received less than 85% of pre-planned chemotherapy schedule. However, the percentage of patients receiving <85% of the dose has been gradually reduced. Indeed, there was a 26% decrease in dose reduction <85% across successive analyses of a same report (Lyman *et al*, 2003; Shayne *et al*, 2006). One could argue that physicians and quality of care control have assumed that the criterion of Bonadonna *et al* (1995) with a cutoff value of 85% as efficacy predictor was major for the management of early breast cancer patients (Budman *et al*, 1998; Mayers *et al*, 2001; Morrow *et al*, 2002; Ottevanger *et al*, 2002; Shayne *et al*, 2006).

For the first use of FEC100 regimen in the French Adjuvant Study Group (FASG) 05 trial, conducted between 1990 and 1993, no prophylactic use of G-CSF was permitted, and an ANC<2 × 10^9^ l^−1^ led to a treatment interruption of at least 1 week (Bonneterre *et al*, 2005). Thereby, among the 268 patients who received the FEC100 regimen, the mean RDI was 86.1%, and seven cases (2.6%) of febrile neutropaenia occurred. More recently, in the PACS 01 trial initiated in 1997 and comparing 6 FEC100 to 3 FEC100-3D, the mandatory ANC for a subsequent cycle delivery was lowered to 1.5 × 10^9^ l^−1^, whereas an ANC<1.5 × 10^9^ l^−1^ required the use of G-CSF for all subsequent cycles (Roche *et al*, 2006). In this trial, the median RDI was 98 and 99%, respectively, whereas G-CSF was prescribed in 27% of the patients receiving FEC100 compared with 22% of those receiving docetaxel. Noteworthy, only 8.4 and 11.2% of patients, respectively, developed a febrile neutropaenia, showing that the prescription of G-CSF aimed predominantly to facilitate neutrophil recovery and to maintain RDI rather than to prevent secondary infections. The comparison between these two randomised trials highlights that changes in treatment modalities lead to a 12% increase of RDI, whereas the use of G-CSF is multiplied by 35 (Bonneterre *et al*, 2005; Roche *et al*, 2006).

One of the major reasons identified for a reduction in chemotherapy doses was neutropaenia (Silber *et al*, 1998; Link *et al*, 2001). Indeed, chemo-induced neutropaenia are often controlled by dose reductions, or cycle delays, which may compromise the disease outcome. The most frequently used option is the use of G-CSF, according to the International Oncology guidelines, which consider that a G-CSF prophylaxis can be used to maintain adequate dose intensity for disease outcome (Greil and Jost, 2005; Aapro *et al*, 2006; Smith *et al*, 2006; Lyman and Kleiner, 2007). However, the guidelines are a little variable on their indications for use of haematopoietic support. For instance, Smith *et al* (2006) did not recommend use maintaining dose intensity for adjuvant breast cancer chemotherapy, as they considered that there is no evidence that a slight decrease in dose or a slight prolongation of dose interval worsens outcome. Moreover, an extension in the use of G-CSF may be counterbalanced by long-term side effects. First, some data suggested that G-CSF might worsen anaemia in patients receiving adjuvant chemotherapy (Papaldo *et al*, 2006). Of major concerns, an increased risk of secondary acute myeloblastic leukaemia (AML) and myelodysplastic syndrome (MDS) has been recently reported (Smith *et al*, 2003; Praga *et al*, 2005; Veyret *et al*, 2006; Hershman *et al*, 2007; Le Deley *et al*, 2007). The relationship between AML/MDS because of haematopoietic support must be cautiously interpreted because of the accumulation of confusing factors. Meanwhile, G-CSF use should not be assumed to be risk free. The G-CSF could be more cost-effectively and cautiously applied if targeted to patients with identified risk factors of febrile neutropaenia instead of severe neutropaenia (Borg *et al*, 2004). Another option could be a prophylactic use of antibiotics (Schröder *et al*, 1999). However, it remains controversial as recently illustrated by comments following publication of a randomised study on this topic (Cullen *et al*, 2005, and correspondence).

Another means to maintain RDI of adjuvant chemotherapy may be to lower ANC threshold for administration of chemotherapy. Our experience showed that no infectious complications occurred among 76 patients who received a full-dose schedule despite an ANC <1.5 × 10^9^ l^−1^. This observation is in agreement with data reported in the literature showing that an ANC <1.5 × 10^9^ l^−1^ at the onset of chemotherapy was not convincingly associated with an increased risk of febrile neutropaenia (Greil and Jost, 2005). Previously, the National Surgical Adjuvant Breast and Bowel Project B-15 and B-16 trials showed that the reintroduction of chemotherapy with ANC ranging between 1.0 and 1.5 × 10^9^ l^−1^ was possible (Fisher *et al*, 1990a, 1990b). In a recent retrospective study involving Hodgkin's lymphoma patients, the ABVD (adriamycin, bleomycin, vinblastine, and dacarbazine) administration irrespective of granulocyte counts allowed the treatment to be given at full dose without delays or significant number of infective episodes (Boleti and Mead, 2007). Authors concluded that they found no increased risk of severe infections despite the vast majority of patients experiencing at least one episode of grades 3–4 neutropaenia, of which one-third had at least one episode of grade 4 toxicity. In the present analysis, neutrophil recovery occurred always around the twenty-first day, and did not justify a postponement of 8 days. We could conclude that the 7-day median cycle delay is a consequence of daily practices based on convenient factors. The reduction of the delay by re-evaluating blood counts after 2–3 days would probably improve RDI.

However, our results must be cautiously considered. First, in our standard practices, an adjuvant chemotherapy was not prescribed to patients older than 70 years outside clinical trials, taking into account that no significant benefits were found in EBCTCG overviews (Early Breast Cancer Trialists' Collaborative Group (EBCTCG), 2005). This standard could be debatable according to recent results of randomised trials showing a significant reduction in breast cancer recurrence and mortality with adjuvant chemotherapy for elderly patients (Fargeot *et al*, 2004; Muss *et al*, 2005). On the other hand, the advanced age has been found to be an independent prognostic factor of decrease in RDI (Bonadonna *et al*, 1995; Crivellari *et al*, 2000; Lyman *et al*, 2003; Shayne *et al*, 2006), and to be associated with a higher rate of neutropaenic complications (Crivellari *et al*, 2000; Dees *et al*, 2000; Muss *et al*, 2007), of which clinical consequences were more severe (Balducci and Yates, 2000). Second, the 3 FEC100-3D regimen may be a favourable schedule for re-treatment with a low ANC as docetaxel (D) induces deep but short neutropaenia always corrected on day 22. A re-treatment at ANC of 1 × 10^9^ l^−1^ or less may not be applied with other adjuvant regimens that affect more strongly bone marrow. It has been found that doxorubicin–CMF regimen necessitated further delays subsequently to the first episode in the absence of dose reduction, dose delay, or G-CSF administration (Rivera *et al*, 2003). Across classical chemotherapy regimens used in clinical practice (Canadian CEF, oral or intravenous CMF, AC), 42% of patients experienced at least one neutropaenic event, and 72% of them developed additional events in subsequent cycles (Chang, 2000). When docetaxel was used concurrently with fluorouracil and cyclophosphamide (TAC) plus systematic antibiotic prophylaxis, the incidence of grades 3–4 neutropaenia on day 21 was 65%, with 24% of febrile neutropaenia requiring subsequent use of G-CSF (Martin *et al*, 2005).

In conclusion, this analysis confirms that four–six cycles of FEC100 and three FEC100-3D regimens are feasible in the general population without severe toxicity. Both regimens provide a high RDI, a worthwhile goal for patients receiving adjuvant chemotherapy for breast cancer. This high RDI was achieved without need for G-CSF, and infectious events were rare in spite of the absence of prophylactic antibiotherapy. Using these regimens, the ANC recovery was always observed after about 3 weeks, without any risk of long-lasting neutropaenia. In our opinion, there is no justification for the historical threshold ANC of 1.5 × 10^9^ l^−1^ for administration of adjuvant chemotherapy.

## Figures and Tables

**Table 1:** Distribution of treatment protocols among the 224 selected breast cancer patients

	**4 FEC100**	**6 FEC100**	**3 FEC100-3D**	**3 FEC100-3D+T**
Patients, *n* (%)	54 (24.1)	59 (26.3)	94 (42.0)	17 (7.6)
				
*Chemotherapy, n* (%)				
Adjuvant	53 (98.1)	37 (62.7)	63 (67.0)	9 (52.9)
Neoadjuvant	1 (1.9)	22 (37.3)	31 (33.0)	8 (47.1)
				
*Age, years*				
Median (range)	49 (30–72)	50 (29–68)	50 (26–69)	48 (31–72)
>60 years, *n* (%)	5 (9.2)	9 (15.2)	20 (21.3)	3 (17.6)
>70 years, *n* (%)	1 (1.8)	0 (0.0)	0 (0.0)	2 (11.8)

D=docetaxel 100 mg m^−2^ every 21 days; FEC100=fluorouracil 500 mg m^−2^, epirubicin 100 mg m^−2^, cyclophosphamide 500 mg m^−2^ every 21 days; T=trastuzumab.

**Table 2:** Relative dose intensity according to treatment protocol

	**4 FEC100**	**6 FEC100**	**3 FEC100-3D (±T)**
Number of patients	54	59	111
RDI, median (range)	99% (75–101)	97% (76–102)	99% (68–102)
RDI>95%, *n* (%)	41 (76)	42 (71)	89 (80)
RDI<85%, *n* (%)	4 (7)	4 (7)	6 (5)

D=docetaxel 100 mg m^−2^ every 21 days; FEC100=fluorouracil 500 mg m^−2^, epirubicin 100 mg m^−2^, cyclophosphamide 500 mg m^−2^ every 21 days; RDI=relative dose intensity; T=trastuzumab.

**Table 3:** Haematologic toxicities of treatment

	**4 FEC100**	**6 FEC100**	**3 FEC100-3D (±T)**	**Total**
Number of patients	54	59	111	224
Patients with toxicity	11	18	22	51
Hospitalisation^a^	1	0	4	5
Changes in treatment plan^b^	1	1	6	8
Cycle delay ⩾7 days	9	16	21	46
Dose reduction ⩾15%	0	4	5	9
G-CSF	2	5	1	8

D=docetaxel 100 mg m^−2^ every 21 days; FEC100=fluorouracil 500 mg m^−2^, epirubicin 100 mg m^−2^, cyclophosphamide 500 mg m^−2^ every 21 days; G-CSF=granulocyte colony-stimulating factor; T=trastuzumab.

a Febrile neutropaenia (*n*=3), cutaneous toxicity (*n*=1), and acute colitis (*n*=1).

b Suppression of one cycle of FEC because of pancytopaenia (*n*=1), suppression of one or two cycles of D because of hypersensitivity (*n*=1) or patient willingness (*n*=2), replacement of one cycle of FEC by D because of severe emesis (*n*=1), replacement of one or two cycles of D by FEC because of cutaneous toxicity (*n*=3).
